# Establishing Virulence Associated Polyphosphate Kinase 2 as a drug target for *Mycobacterium tuberculosis*

**DOI:** 10.1038/srep26900

**Published:** 2016-06-09

**Authors:** Mamta Singh, Prabhakar Tiwari, Garima Arora, Sakshi Agarwal, Saqib Kidwai, Ramandeep Singh

**Affiliations:** 1 Vaccine and Infectious Disease Research Centre, Translational Health Science and Technology Institute, Haryana, India

## Abstract

Inorganic polyphosphate (PolyP) plays an essential role in microbial stress adaptation, virulence and drug tolerance. The genome of *Mycobacterium tuberculosis* encodes for two polyphosphate kinases (PPK-1, Rv2984 and PPK-2, Rv3232c) and polyphosphatases (*ppx-1*, Rv0496 and *ppx-2*, Rv1026) for maintenance of intracellular PolyP levels. Microbial polyphosphate kinases constitute a molecular mechanism, whereby microorganisms utilize PolyP as phosphate donor for synthesis of ATP. In the present study we have constructed *ppk-2* mutant strain of *M. tuberculosis* and demonstrate that PPK-2 enzyme contributes to its ability to cause disease in guinea pigs. We observed that *ppk-2* mutant strain infected guinea pigs had significantly reduced bacterial loads and tissue pathology in comparison to wild type infected guinea pigs at later stages of infection. We also report that in comparison to the wild type strain, *ppk-2* mutant strain was more tolerant to isoniazid and impaired for survival in THP-1 macrophages. In the present study we have standardized a luciferase based assay system to identify chemical scaffolds that are non-cytotoxic and inhibit *M. tuberculosis* PPK-2 enzyme. To the best of our knowledge this is the first study demonstrating feasibility of high throughput screening to obtain small molecule PPK-2 inhibitors.

Inorganic polyphosphate (PolyP) is a linear polymer of inorganic phosphate linked by phosphoanhydride bond^1^. PolyP is ubiquitously present in all domains of life (archaea, bacteria and eukarya) and plays an important role in various cellular physiological functions^2^,^3^,^4^. These functions include processes such as substitute for ATP in enzymatic reactions, chelator of divalent metal ions, phosphate reservoir and microbial adaptation to numerous stress conditions^2^,^3^,^4^,^5^,^6^. In bacterial pathogens, polyphosphate kinase -1 (PPK-1) catalyzes the reversible transfer of the terminal phosphate group of ATP to form long chain polyphosphates and the exopolyphosphatase (PPX) enzyme cleaves the phosphoanhydride bonds of PolyP to generate inorganic phosphate^7^,^8^,^9^. The intracellular PolyP levels in bacteria fluctuate upon its exposure to various stress conditions and decrease in PolyP levels is associated with impairment of several important structural and cellular functions such as motility, quorum sensing, biofilm formation and virulence^4^,^10^,^11^,^12^,^13^,^14^,^15^,^16^,^17^,^18^,^19^. In addition to its PolyP synthetic activity, PPK-1 also utilizes PolyP as phosphate donor for synthesis of nucleoside triphosphates from nucleoside diphosphates. In case of *E. coli* PPK-1, the order of substrate specificity for reverse reaction is ADP>GDP>CDP or UDP^20^.

In addition to PPK-1, another widely conserved enzyme in PolyP metabolism is polyphosphate kinase 2 (PPK-2) enzyme^21^. PPK-2 enzyme contains motif for phosphate binding (P-loop, GXXXXGK) and is broadly classified into three subfamilies, class I, II and III based on their substrate specificity^22^. Class I and II PPK-2 enzymes catalyze nucleoside diphosphate and nucleoside monophosphate phosphorylation whereas class III PPK-2 enzyme is able to synthesize nucleoside triphosphates directly from nucleoside monophosphates^22^,^23^,^24^,^25^. PPK-2 from *Pseudomonas aeruginosa* is upregulated by >100 fold during stationary phase and PPK-2 derived GTP is required for alginate production, the exopolysaccharide that envelopes the bacteria^23^,^26^. In addition, PPK-2 has also been demonstrated to mediate an important role in stress tolerance and pathogenesis of *Campylobacter jejuni*^25^.

*M. tuberculosis* genome harbors enzymes involved in both PolyP synthesis (PPK-1, Rv2984) and its utilization (PPK-2, Rv3232c and PPX, Rv0496 and Rv1026^27^). *In vitro* quantification experiments revealed that mycobacteria accumulates PolyP at later stage of growth, upon exposure to stress conditions such as oxidative, nitrosative, nutritional, low oxygen and drugs such as rifampicin (Rif), levofloxacin (Levo), Isoniazid (Inh) and Gentamycin (Gm)^28^,^29^. Several studies demonstrate that any dysregulation in PolyP levels is associated with impaired survival of *M. tuberculosis* in macrophages and guinea pigs^29^,^30^. We have also previously shown that PolyP deficiency is associated with increased susceptibility of *M. tuberculosis* to front-line TB drugs^29^. PPK-2 enzyme from *M. tuberculosis* has been biochemically characterized, forms an octameric multimer, undergoes auto-phosphorylation and rates of PolyP dependent ATP synthesis are approximately 800 folds greater than the rates of PolyP synthesis^31^. In another study it has been shown that PPK-2 homolog regulates intracellular nucleoside triphosphate levels by interacting with Nucleoside diphosphate kinase A (NdkA) and contributes to *M. smegmatis* adaptation to conditions such as heat, acidic or hypoxia^32^.

In this study, we have cloned, expressed and purified *M. tuberculosis* PPK-2 enzyme for further biochemical characterization. We have constructed *ppk-2* mutant strain of *M. tuberculosis* using temperature sensitive mycobacteriophages and compared the growth of various strains *in vitro*, in macrophages and guinea pigs. We demonstrate that PPK-2 enzyme is important for *M. tuberculosis* to establish disease in guinea pigs. Subsequently, we have screened a small molecule library and identified novel chemical scaffolds that are non-cytotoxic and inhibit PPK-2 enzyme in a dose dependent manner.

## Results

### Biochemical characterization of *M. tuberculosis* PPK-2

**Figure 1:**
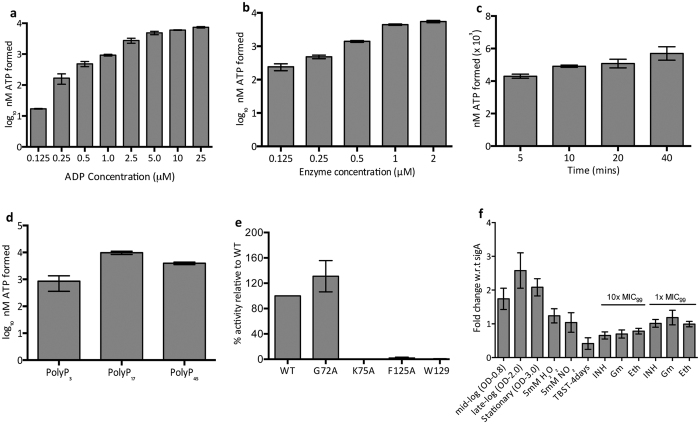
(**a**) PPK-2 activity assays using various concentrations of ADP. For activity assays PPK-2 was expressed and purified as NH_2_-terminus MBP-tagged fusion protein. The enzyme reaction was performed using 1 μM MBP-PPK-2 in the presence of 10 μM PolyP_17_ and 0.125 to 25 μM ADP at 37 °C for 10 minutes. Each value on y-axis represents mean ± S.E. of log_10_ nM ATP obtained from three independent enzyme reactions. (**b**) PPK-2 reaction was performed in the presence of 10 μM PolyP_17_ and 10 μM of ADP at 37 °C for 10 minutes using different concentration of enzyme (0.125 to 2 μM). The data presented on y-axis represents mean ± S.E. of log_10_ nM ATP values obtained from 3 independent enzymatic reactions. (**c**) PPK-2 reaction was performed in the presence of 10 μM PolyP and 10 μM ADP at 37 °C using 1 μM MBP-PPK2. The amount of ATP generated was measured at different time intervals of enzymatic assays. The data presented on y-axis represents mean ± S.E. nM ATP formed in enzymatic assays from three independent experiments. (**d**) The enzyme reaction was performed using 1 μM MBP-PPK-2 in the presence of either 10 μM PolyP_3_, PolyP_17_ or PolyP_45_ and 10 μM ADP at 37 °C for 10 minutes. Each value on y-axis represents mean ± S.E. of log_10_ nM ATP values obtained from three independent enzymatic reactions. (**e**) Enzymatic assays were performed using 1 μM of either wild type or mutant PPK-2 enzymes in the presence of 10 μM PolyP_17_ and 10 μM ADP at 37 °C for 10 mins. Data depicted on y-axis represents mean ± S.E. of percentage activity relative to wild type protein obtained from triplicate experiments. (**f**) qRT-PCR studies to measure *ppk-2* transcript levels under various growth and stress conditions. mRNA was isolated from *M. tuberculosis* cultures at various growth stages and early-log phase cultures exposed to various stress conditions/drugs. The data presented in this panel is mean ± S.E. for fold change in *ppk-2* transcript levels in mid-log, late-log, stationary phase and after exposure to various stress conditions/drugs relative to its levels in early-log phase growth conditions (OD_600nm_-0.3).

*M. tuberculosis* PPK-2 enzyme belongs to P-loop kinases superfamily and possesses both highly conserved Walker A and Walker B motif^21^,^24^ (Fig. S1). The Walker A motif (or P-loop, GXXXXGK) binds the β and γ phosphates of ATP whereas the conserved Asp residue in Walker B motif (DRS) coordinates with Mg^2+^ ion bound to β and γ phosphates residues of ATP. For biochemical characterization, the *ppk-2* gene was PCR amplified, cloned into pMAL-c2x and purified as MBP-tagged protein using amylose resin as per manufacturer’s recommendations. The purified MBP-PPK-2 fractions were pooled, dialyzed, concentrated and assayed for PPK-2 activity. In our enzymatic assays we observed that MBP-PPK-2 possesses Ndk-like activity utilizing PolyP as the phosphate donor for ATP synthesis. We show that ATP synthetic activity of PPK-2 increased in the presence of both ADP and enzyme in a dose dependent manner (Fig. 1a,b). As shown in Fig. 1c, majority of ATP synthesis by PPK-2 was attained in initial 5 minutes of the enzymatic reaction. In contrast to purified PPK-1 enzyme, MBP-PPK-2 could also utilize short chain polyphosphates, PolyP_3_ as the phosphate donor, however, its activity increased in the presence of long chain polyphosphates PolyP_17_ or PolyP_45_^29^ (Fig. 1d). It has been demonstrated that PPK-2 enzyme undergoes autophosphorylation and these residues (His^115^, His^247^) are essential for activity^32^. For mutational analysis in this study Gly^73^ and Lys^75^ located in the P-loop motif were selected. Besides these, the highly conserved Phe^125^ and Trp^129^ residues surrounding the Walker B motif were also shortlisted for mutational studies. We next compared the activity of wild type and mutant PPK-2 enzymes in our enzymatic assays (Fig. 1e). We observed that purified MBP-K75APPK-2, MBP-F125APPK-2 and MBP-W129APPK-2 proteins had significantly reduced activity as compared to wild type protein (Fig. 1e). However the activity of MBP-G72APPK-2 and wild type proteins were similar in our enzymatic assays (Fig. 1e).

### Expression of PPK-2 during different *in vitro* conditions

Identifying the conditions under which PPK-2 is upregulated is essential to understand its role in *M. tuberculosis* physiology. In the present study we have performed quantitative real time PCR (qRT-PCR) to measure *ppk-2* transcript levels under various growth conditions and upon exposure to multiple stress conditions *in vitro* (Fig. 1f). We observed that *ppk-2* transcripts were upregulated by 2.0–2.5 fold during mid-log, late-log and stationary phase stages of *M. tuberculosis* growth in liquid cultures (Fig. 1f). However, a marginal decrease in *ppk-2* transcript level was observed upon exposure of *M. tuberculosis* to nutritional stress. We also report that exposure to either oxidative or nitrosative stress conditions had no effects on *ppk-2* transcript levels (Fig. 1f). We next investigated the effects of drug exposure on *ppk-2* transcription and observed exposure of *M. tuberculosis* to both 10x and 1x MIC_99_ concentration of isoniazid, gentamycin and ethambutol had minimal effects on *ppk-2* transcript levels (Fig. 1f).

### Construction and characterization of *ppk-2* mutant strain

**Figure 2:**
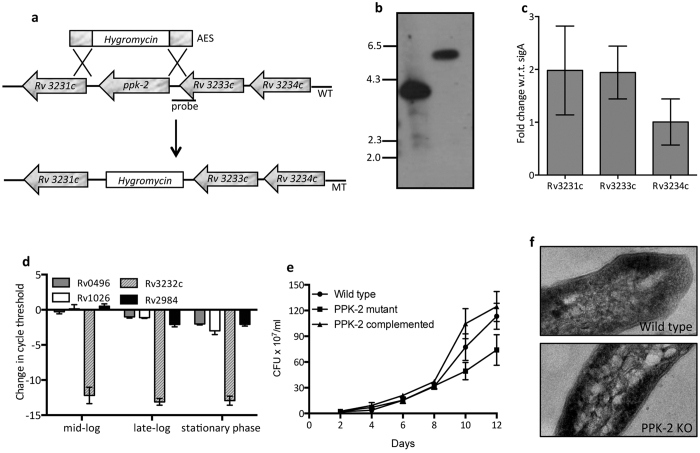
(**a**) Schematic representation of *ppk-2* locus in wild type (WT) and *ppk-2* mutant (MT) strains of *M. tuberculosis*. Details of the *ppk-2* locus in the genome of WT and MT strains of *M. tuberculosis*. In MT strain, *ppk-2* has been replaced by hygromycin resistance gene using temperature sensitive mycobacteriophages as per standard protocols. (**b**) Southern Blot analysis. Genomic DNA was isolated from various strains using CTAB method. 5 μg of genomic DNA was digested with *Bam* HI, transferred to Hybond N membrane and probed with *ppk-2* locus specific DIG labeled probe as shown in panel (**a**). **(c,d**) Effect of *ppk-2* deletion on transcription of *ppk-2* neighboring genes and other enzymes involved in PolyP metabolism. qRT-PCR of *ppk-2* neighboring genes ((**c**) Rv3233c, Rv3234c and Rv3231c) and PolyP metabolic enzymes ((**d**), *ppk-1*, Rv2984, *ppk-2*, Rv3232c, *ppx*, Rv0496 and Rv1026) was performed using mRNA isolated from mid-log, late-log and stationary phase cultures of wild type and *ppk-2* mutant strains. Data shown is change in cycle threshold in the *ppk-2* mutant strain relative to that in wild type strain obtained from three independent experiments. (**e**) Influence of *ppk-2* deletion on growth of *M. tuberculosis in vitro*. Growth characteristics of wild type, *ppk-2* mutant and *ppk-2* complemented strain was evaluated by measuring CFU/ml. (**f**) Transmission electron microscopy images of wild type and *ppk-2* mutant strain show that cell wall integrity was comparable from late-log phase cultures of these strains.

To investigate the role of PPK-2 enzyme in *M. tuberculosis* stress adaptation and virulence, we constructed *ppk-2* mutant strain using temperature sensitive mycobacteriophages^33^ (Fig. 2a). The replacement of open reading frame of PPK-2 enzyme with hygromycin resistance gene in the genome of *ppk-2* mutant strain was confirmed by Southern blot and qRT-PCR studies (Fig. 2b,d). In a recent study by Chuang *et al*, it has been shown that *ppk-2* is co-transcribed along with its upstream genes, Rv3233c and Rv3234c and genes downstream to *ppk-2* (Rv3231c and Rv3230c) are co-transcribed independently in a single transcript^34^. To design our strategy for construction of complemented strain, qRT-PCR was performed to determine whether replacement of *ppk-2* with hygromycin resistance gene had any polar effect on transcription of its neighboring genes (Fig. 2c). In concordance with previous reports, Rv3231c, Rv3233c and Rv3234c transcript levels were comparable in both wild type and *ppk-2* mutant strain, thereby suggesting lack of polar effect in the mutant strain^34^. The *ppk-2* complemented strain was constructed by re-introducing a copy of *ppk-2* in trans using an integrative expression vector pJEB402 to yield *ppk-2* complemented strain. The restoration of *ppk-2* transcript levels in *ppk-2* complemented strain was verified by qRT-PCR (data not shown). We also compared the transcript levels of *ppk-1* (Rv2984) and *ppx* (Rv0496 and Rv1026) enzymes in both wild type and *ppk-2* mutant strain at different stages of growth (Fig. 2d). Similar to previous studies we observed that transcript levels of *ppk-1* and *ppx* were reduced in *ppk-2* mutant strain relative to wild type strain at late-log and stationary phase growth conditions (Fig. 2d). However, the transcript levels for these PolyP metabolic enzymes remained unaltered in both wild type and *ppk-2* mutant strain during mid-log stage of growth^34^ (Fig. 2d).

Numerous studies have shown that bacterial strains harboring mutations in PolyP metabolic enzymes display altered growth defect in liquid medium, colony morphology and biofilm formation. As shown in Fig. 2e, the *ppk-2* mutant strain had a slight growth defect (~2.0 fold) in liquid medium and we were able to restore this defect in the *ppk-2* complemented strain. In this study we observed that wild type, *ppk-2* mutant strain and *ppk-2* complemented strains displayed similar colony morphology in solid medium and biofilm formation in detergent free Sauton’s medium (Fig. S2a,b). In a study by Chuang *et al*, TEM analysis revealed that *M. tuberculosis* strains accumulating PolyP are shorter in length as compared to wild type bacilli^28^. However, in our study, we observed that the cell wall architecture and bacterial length of wild type and *ppk-2* mutant strain were similar to each other (Fig. 2f).

### PPK-2 is associated with reduced INH susceptibility and required for intracellular survival in macrophages

**Figure 3:**
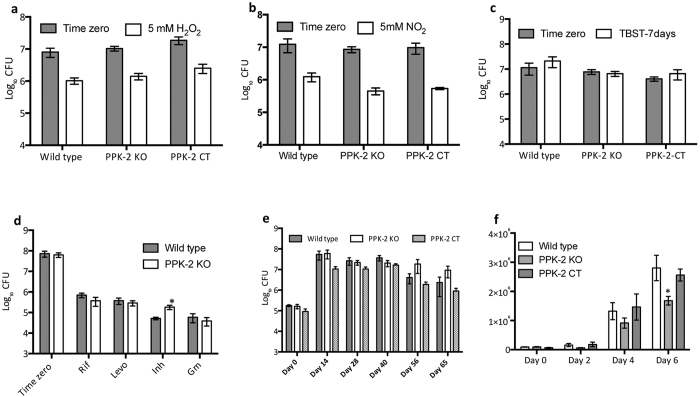
(**a–d**) Susceptibility of various *M. tuberculosis* strains to stress conditions and drugs *in vitro*. Early-log phase cultures of wild type and *ppk-2* mutant strains were exposed to either 5 mM H_2_O_2_ for 24 hours (**a**) or 5 mM NaNO_2_ at pH 5.2 for 3 days (**b**) and TBST for 7 days (**c**). For *in vitro* drug tolerance experiments (**d**), mid-log phase cultures of various strains were exposed to various drugs with different mechanism of action. The concentration of drugs used in the experiments was Rif (0.4 μg/ml), Levo (10 μg/ml), Gm (10 μg/ml) and Inh (10 μg/ml). Bacterial enumeration was performed by plating 100 μl of 10-fold serial dilution on MB-7H11 plates and incubating the plates at 37 °C for 3–4 weeks. Data shown in these panels are mean ± S.E. obtained from three independent experiments. Significant differences were observed for indicated groups, paired (two-tailed) t-test P < 0.05. (**e**) Effect of PPK-2 on *M. tuberculosis* growth in non-replicative persistence stage. Non-replicative hypoxia stage was generated in sealed tubes. Bacterial enumeration was performed at day 14, day 28, day 40, day 56 and day 65 by plating 10.0-fold serial dilutions. Data shown in mean ± S.E. obtained from 2 independent experiments. (**f**) Influence of *ppk-2* gene disruption on *M. tuberculosis* growth in THP-1 macrophages. THP-1 macrophages were infected with either wild type or *ppk-2* mutant or *ppk-2* complemented strain at an MOI of 1:1. At day 0, 2, 4 and 6 post-infection macrophages were washed and lysed in 1× PBS containing 0.1% Triton X-100 and bacterial counts were determined. Data depicted in this panel is mean ± S.E. obtained from two independent experiments performed in triplicate wells. Significant differences were observed for indicated groups, paired (two-tailed) t-test P < 0.05.

In *Campylobacter jejuni*, deletion of *ppk-2* impairs its ability to survive upon exposure to nutritional and osmotic stress^25^. *M. tuberculosis* is a extremely successful intracellular pathogen by virtue of its ability to sense and adapt to diverse range of stress conditions such as low oxygen, acidic pH and nutrient deprivation. Therefore, we next compared the survival rates of wild type, *ppk-2* mutant and *ppk-2* complemented strain upon exposure to various stress conditions such as oxidative, nitrosative, nutritional, low oxygen and in THP-1 macrophages (Fig. 3). We observed that *ppk-2* mutant strain was comparable to wild type strain in its ability to survive upon exposure to either oxidative, nitrosative or nutritional stress (Fig. 3a–c).

Consistent with previously published reports, we observed that intracellular PolyP levels are elevated in *ppk-2* mutant strain in comparison to wild type and *ppk-2* complemented strains^34^ (data not shown). We had earlier reported that PolyP deficiency is associated with enhanced susceptibility of *M. tuberculosis* to front-line TB drugs^29^. Therefore, we next investigated whether PolyP accumulation in *ppk-2* mutant strain is associated with enhanced tolerance against front-line TB drugs. We observed that as compared to the wild type strain, *ppk-2* mutant strain was approximately 3.0–4.0 fold more tolerant upon exposure to cell wall inhibitor, INH (Fig. 3d, p < 0.05). However, the percentage persisters were similar for both wild type and *ppk-2* mutant strain upon exposure to other known TB drugs such as Rif, Levo or Gm (Fig. 3d). These results suggest that various metabolic pathways contribute to the phenomenon of persistence in a drug-specific manner. As shown in Table S1, minimum inhibitory concentration (MIC_99_) values for various drugs tested in the study were comparable for both wild type and *ppk-2* mutant strain. In order to understand the role of PPK-2 enzyme in *M. tuberculosis* adaptation to low oxygen conditions, we next compared the growth of wild type, *ppk-2* mutant and *ppk-2* complemented strains in hypoxia. We observed that the bacterial numbers were 3.0–4.0 fold more in the case of *ppk-2* mutant strain in comparison to wild type strain at day-56 and day-65 in Wayne hypoxia model (Fig. 3e). The bacterial numbers were restored in the *ppk-2* complemented strain upon reintroduction of PPK-2 expression in the mutant strain (Fig. 3e). To investigate the role of PPK-2 in intracellular survival of *M. tuberculosis*, THP-1 macrophages were infected with either wild type or *ppk-2* mutant or *ppk-2* complemented strain at an MOI of 1:1. As shown in Fig. 3f, we observed that wild type, *ppk-2* mutant and *ppk-2* complemented strain displayed similar growth kinetics within macrophages upto 4 days post-infection. However, *ppk-2* mutant strain displayed a growth defect of 2.0-fold in comparison to the wild type strain and *ppk-2* complemented strain at day 6 post-infection (Fig. 3f, p < 0.05).

### The *ppk-2* mutant strain of *M. tuberculosis* is attenuated for growth *in vivo*

**Figure 4:**
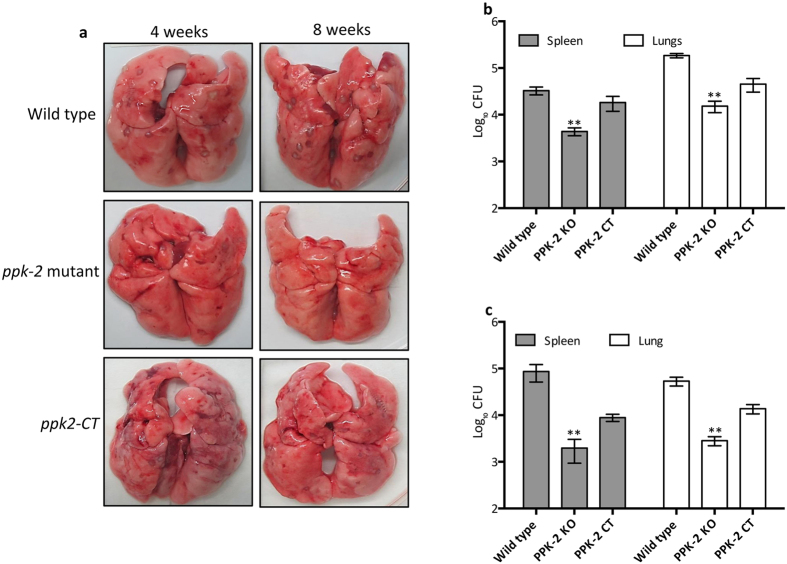
Effect of *ppk-2* deletion on *M. tuberculosis* growth in guinea pigs. (**a**) The panel depicts representative lung images from 4 weeks or 8 weeks infected guinea pigs. (**b,c**) The panel depicts lungs and splenic bacterial loads of aerosol infected guinea pigs at 4 weeks (**b**) and 8 weeks (**c**) post-infection. The data represented in this panel is mean ± S.E. log_10_ values obtained for each group (6–7 animals/group). Significant differences were observed for indicated groups, paired (two-tailed) t-test P < 0.01.

Next, we determined the role of PPK-2 in *M. tuberculosis* ability to grow and persist *in vivo* by infecting guinea pigs with various strains. For these experiments, guinea pigs were infected via aerosol route with log_10_ 1.93 ± 0.06, log_10_ 2.13 ± 0.1, log_10_ 2.06 ± 0.1 (these values are log_10_ number of CFU/lung) delivered in the case of wild type, *ppk-2* mutant and *ppk-2* complemented strains, respectively. Gross pathology analysis revealed that lung tissues from wild type and *ppk-2* complemented strains infected groups showed discrete multiple large tubercles, whereas their numbers and size were fewer in *ppk-2* mutant infected guinea pigs (Fig. 4a). However, the total lung weight from wild type, *ppk-2* mutant and *ppk-2* complemented strains infected guinea pigs were almost identical (data not shown). As expected *M. tuberculosis* growth was observed in all three groups, while lungs infected with wild type bacteria had 12.0 fold higher bacterial numbers in comparison to lungs infected with *ppk-2* mutant strain at 4 weeks post-infection (Fig. 4b, p < 0.01). Similarly, the splenic bacterial counts in *ppk-2* mutant infected guinea pigs (log_10_ 3.6 ± 0.88) were significantly lower by 7.5 fold as compared to wild type infected guinea pigs (log_10_ 4.5 ± 0.084, p < 0.01). The *ppk-2* mutant strain exhibited a more dramatic growth defect of 18.5 folds and 42.0 folds in lungs and spleens, respectively at day-56 post-infection (Fig. 4c, p < 0.01). As shown in Fig. 4b,c, we observed that bacterial loads in tissues from *ppk-2* complemented strains were increased to those observed in *ppk-2* mutant infected guinea pigs at both 4 weeks and 8 weeks post-infection.

**Figure 5:**
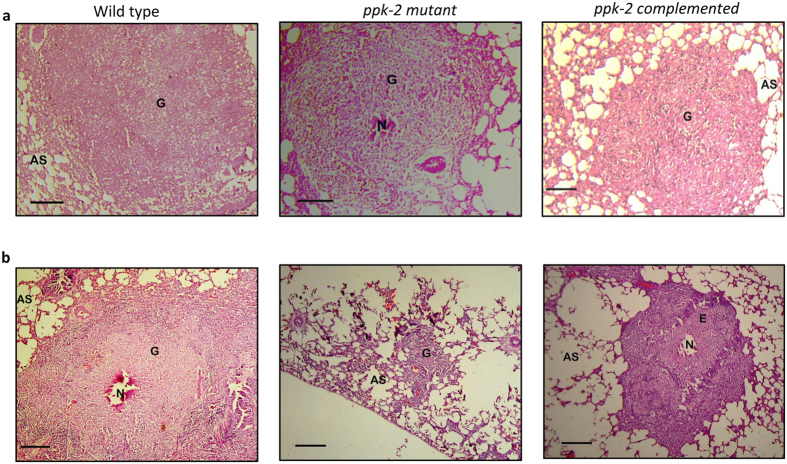
Histopathological analysis of lung sections of infected guinea pigs. The images depicts representative 40x photomicrographs of hematoxylin and eosin (H & E) stained 5 μm lung sections of guinea pigs infected with various strains and euthanized at 4 (**a**) and 8 (**b**) weeks post-infection. AS, E, G and N represent alveolar space, epitheloid cells, granulomas and necrosis, respectively.

**Figure 6:**
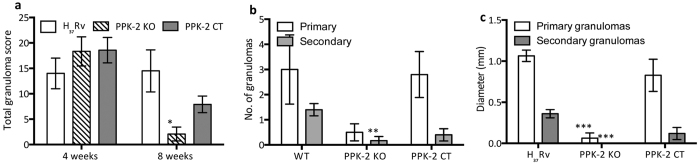
Quantitative analysis of tissue damage of lung sections of guinea pigs infected with various strains. (**a**) Total granuloma score (mean ± S.E.) in H&E stained lung sections of animals infected with various strains (5–6 guinea pigs per group) at 4 weeks and 8 weeks post-infection. Significant differences were observed for indicated groups, paired (two-tailed) t-test P < 0.05. (**b**) The figure depicts mean ± S.E. of number of primary and secondary granulomas in lung sections of animals infected with various strains at 8 weeks post-infection. Significant differences were observed for indicated groups, paired (two-tailed) t-test P < 0.01. (**c**) The panel depicts mean ± S.E. of diameter of primary and secondary granulomas in lung sections of animals infected with various strains at 8 weeks post-infection. Significant differences were observed for indicated groups, paired (two-tailed) t-test P < 0.001.

In agreement with these growth patterns we observed similar extent of tissue damage with large granulomas in the lung parenchyma from all three groups at 4 weeks post-infection (Fig. 5). Majority of these lung granulomas were composed primarily of epitheloid cells with peripheral lymphocytic rich zones while few granulomas showed necrotic centers with acute inflammatory cellular infiltration in and around the necrotic areas (Fig. 5a). A more detailed quantitative analysis revealed that the total granuloma scores in lung sections from guinea pigs infected with the wild type and *ppk-2* mutant were 14.0 and 19.0 respectively at 4 weeks post-infection (Fig. 6a). As expected, at 8 weeks post-infection significantly reduced tissue damage was observed in lung tissue sections from *ppk-2* mutant infected guinea pigs as compared to wild type and *ppk-2* complemented infected guinea pigs (Fig. 5b). As shown in Fig. 6a, total granuloma scores in lung sections of wild type infected guinea pigs were significantly higher by ~7.0 fold in comparison to those obtained in sections from *ppk-2* mutant infected guinea pigs at 8 weeks post-infection (p < 0.05). The section from wild type infected guinea pigs displayed large granulomas with central necrosis whereas *ppk-2* mutant infected sections showed granulomas lacking necrotic centers and comprised mainly of epitheloid cells and lymphocytes. We observed that both number and diameter of granulomas (primary and secondary) were higher in lung sections from wild type infected guinea pigs in comparison to sections from guinea pigs infected with *ppk-2* mutant strain (Fig. 6b, p < 0.01 and 6c, p < 0.001). The percentage of surface area involved by inflammation in lung sections from wild type infected guinea pigs (15.80 ± 6.2) was significantly higher in comparison to lung sections from *ppk-2* infected guinea pigs (0.833 ± 0.54) at 8 weeks post-infection (p < 0.01). We also observed that genetic complementation of *ppk-2* mutant strain with wild type copy under the control of *hsp60* promoter increased lung involvement, thereby, indicating attenuation observed *in vivo* was due to disruption of *ppk-2* in *M. tuberculosis*.

### Identification of PPK-2 inhibitors using high throughput approach

**Figure 7:**
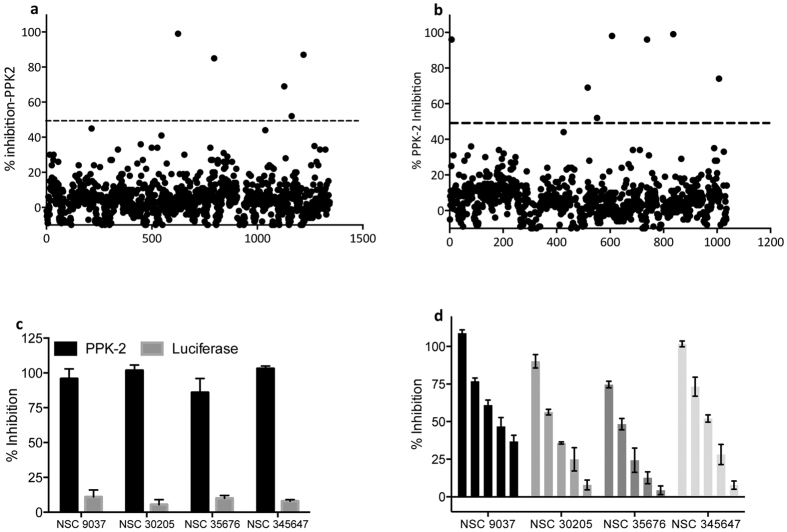
Highthrough put screening using NCI-DTP library to identify novel PPK-2 small molecule inhibitors. (**a,b**) NCI-DTP library comprising of 2300 compounds belonging to either diversity (**a**) or mechanistic and natural product (**b**) was evaluated to identify PPK-2 inhibitors. Data presented in panels (**a,b**) is percentage inhibition obtained from two independent experiments. (**c**) PPK-2 and luciferase enzyme inhibition by primary hits. PPK-2 and luciferase based assay systems were performed in the presence of primary hits as described in Methods. The values on y-axis depicted in this panel is percentage inhibition obtained from three independent experiments. (**d**) PPK-2 inhibition assays were performed in the presence of either NSC 9037 or NSC 30205 or NSC 35676 or NSC 345647 at concentrations ranging from 100 to 6.25 μM. The percentage inhibition values are depicted on y-axis and are mean ± S.E. obtained from three independent experiments.

**Figure 8:**
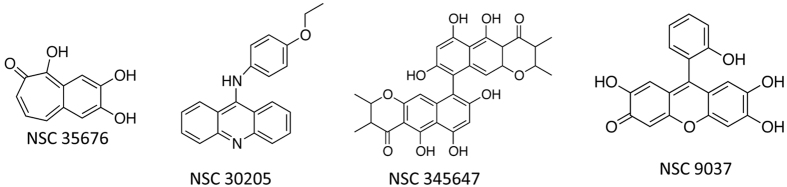
Chemical structures of PPK-2 specific inhibitors identified from HTS assays.

In order to identify PPK-2 inhibitors, an endpoint assay system was developed that is amenable to 96-well format for high throughput screening. For initial standardizations, various parameters such as buffer pH, ion concentration and reaction time were investigated and optimum PPK-2 activity was observed in assay conditions; 50 mM Tris-Cl pH-7.4, 10 mM (NH_4_)_2_SO_4_ and 10 mM MgCl_2_ after 20 minutes of incubation at 37 °C (data not shown). NCI-DTP library comprising of 2300 compounds belonging to either diversity set, mechanistic set or natural product set was screened to identify novel inhibitors for PPK-2 enzyme. Preliminary screening at 100 μM concentration yielded 12 compounds with a hit rate of 0.5% that inhibited PPK-2 activity by >50%. As shown in Fig. 7a,b seven compounds inhibited PPK-2 activity by >50% and five compounds displayed 50–75% inhibition of PPK-2 activity. However, in our repeat inhibition experiments only 10 scaffolds inhibited PPK-2 dependent ATP synthesis by >50%. Cross-reactivity of these inhibitors with luciferase enzyme would be a major concern in further validation of these primary hits as PPK-2 inhibitors. Therefore, we compared the ability of these identified hits to inhibit luciferase and PPK-2 enzyme *in vitro* at 100 μM concentration. We observed that six out of these 10 compounds inhibited both luciferase and PPK-2 enzyme to similar extent (data not shown). As shown in Fig. 7c remaining 4 compounds NSC-35676, NSC-30205, NSC-345647 and NSC-9037 specifically inhibited PPK-2 enzyme by >80% at 100 μM concentration. As expected, these scaffolds inhibited PPK-2 enzyme in our *in vitro* assays in a dose dependent manner (Figs 7d and 8).

In our WST-1 based cell viability assays, NSC9037 and NSC 35676 were non-cytotoxic to THP-1 cells even at 50 μM concentration (highest concentration tested in the study). Remaining two scaffolds NSC-35647 and NSC-30205 were cytotoxic against THP-1 macrophages with TC_50_ values of 5 and 10 μM, respectively. The results presented in this study demonstrate that PPK-2 is an *in vivo* drug-target and contributes to *M. tuberculosis* ability to cause disease in guinea pigs. The 96-well standardized luciferase based assay system could be explored further to identify more potent PPK-2 specific non-cytotoxic inhibitors.

## Discussion

*M. tuberculosis* is a extremely successful intracellular pathogen and possesses various virulence determinants such as extra-cytoplasmic sigma factors and two component systems that enable it to evade host immune response and adapt to various stress conditions encountered in the host^35^,^36^. PolyP, a linear polymer of inorganic phosphate accumulates in *M. tuberculosis* upon stress and drug-exposure *in vitro*^28^,^29^. The genome of *M. tuberculosis* harbors enzymes involved in both PolyP synthesis and utilization. PPK-1, PPK-2 and PPX have been biochemically characterized and in comparison to the parental strain, *ppk-1* and *ppx* mutant *M. tuberculosis* strains are attenuated for growth in guinea pigs^28^,^29^. In the present study we have performed experiments to i) understand the role of PPK-2 enzyme in *M. tuberculosis* physiology and virulence and ii) to identify small molecule inhibitors for PPK-2 enzyme. In this study, we report that *M. tuberculosis* PPK-2 belongs to class-I subfamily and is able to utilize PolyP of different chain lengths as phosphate donor for synthesis of ATP. In addition to previously identified essential amino acids His-115, His-245 and Gly-74, we report that mutation of Lys-75, Phe-125 and Trp-129 significantly reduced the activity of PPK-2 enzyme *in vitro*^32^.

Previous studies have demonstrated that PPK-2 enzyme interacts with NdkA enzyme to maintain intracellular GTP levels in *M. smegmatis*. The *ppk-2* mutant of *M. smegmatis* displayed increased ATP/GTP ratios and was compromised in its ability to survive under conditions such as heat or acidic or hypoxia^32^. Concordantly, the ATP/GTP ratios were also marginally increased in *ppk-2::tn* of *M. tuberculosis* in comparison to the wild type strain^34^. In this study we have performed experiments to understand the significance of PPK-2 in stress adaptation and virulence using guinea pigs. The *ppk-2* mutant strain was constructed using temperature sensitive mycobacteriophages and it displayed a slight growth defect in stationary phase as compared to the wild type strain. We observed that increase in ATP/GTP and PolyP levels is associated with enhanced tolerance of *M. tuberculosis* towards INH. This tolerance phenomenon was INH specific as both wild type and *ppk-2* mutant strains were equally susceptible to other drugs evaluated in the study. The observed reduced susceptibility towards INH was due to presence of INH-tolerant persisters and not due to emergence of INH-resistant mutant strains. This INH tolerant phenotype is consistent with enhanced and reduced INH susceptibility phenotype observed for PolyP deficient (*ppk-1*) and PolyP accumulating (*ppx*) mutant strains of *M. tuberculosis*, respectively^29^,^30^. We also observed that both wild type and *ppk-2* mutant strain of *M. tuberculosis* were susceptible to similar levels upon exposure to other stress conditions *in vitro*.

The low-dose aerosol guinea pig model was used to determine the effect of *ppk-2* deletion on *M. tuberculosis* growth *in vivo*. Although, the *ppk-2* mutant was implanted at a higher dose, the number of bacteria were 12.0 and 7.5 folds lower in lungs and spleens, respectively, in comparison to wild type infected guinea pigs at 4 weeks post-infection. This survival defect increased to approximately 18.5 fold and 42.0 fold in lungs and spleens, respectively at day 56 post-infection. We observed incomplete restoration of wild type phenotype following complementation of *ppk-2* mutant strain with wild type copy of *ppk-2*. Histological analysis were in concordance with bacterial burdens as fewer granulomas were observed in *ppk-2* mutant infected guinea pigs as compared to wild type and *ppk-2* complemented guinea pigs at 8 weeks post-infection. The growth defect associated with the *in vivo* survival of *ppk-2* mutant strain was more apparent in guinea pigs in comparison to mouse infection studies^34^. A possible explanation for these contradicting findings could be use of different animal models for respective virulence studies. The guinea pigs following *M. tuberculosis* infection form classically appearing caseous granulomas whereas granulomas observed in mouse are diffuse^37^. In addition, similar to human tuberculosis lesions, the oxygen levels are profoundly reduced in guinea pig granulomas in comparison to mouse granulomas. Another variation in these studies was the nature of mutant strains used for animal experiments. Chaung *et al*, utilized *ppk-2:Tn* mutant for their mice infection studies, whereas in our guinea pig experiments we have employed a strain in which PPK-2 open reading frame has been replaced by hygromycin resistance gene^34^. However, a limitation of our study is the lack of cytokine data that would enable us to understand the contribution of host immune response to control infection caused by *ppk-2* mutant strain of *M. tuberculosis*. Our findings are similar to other studies where survival phenotypes associated with mutant strains are more apparent in guinea pigs as compared to mice^38^,^39^. The observed survival defect of *ppk-2* mutant strain is similar to the phenotype observed for another PolyP accumulating strain deficient in exopolyphosphatase, Rv0496^30^. These observations suggest that PPK-2 contributes to *M. tuberculosis* adaptation to conditions implicated by the host immune response specifically in guinea pigs. Similar to our observations, *ppk-2* deletion is also associated with survival defect of *Campylobacter jejuni* within human intestinal epithelial cells^25^.

Despite identification and validation of various novel drug targets and scaffolds, a major challenge in the field of chemotherapy is emergence of various drug-resistant TB strains^40^. Therefore, validation of newer *in vivo* drug targets and identification of scaffolds with novel mechanism of action is urgently required. These identified newer scaffolds should i) target heterogeneous bacterial population ii) target MDR and XDR *M. tuberculosis* strains iii) be compatible with the current TB regimen and iv) shorten the duration of lengthy TB chemotherapy^41^. Recently, FDA has approved bedaquiline, a drug that target *M. tuberculosis* ATP synthase for treatment of MDR-TB patients^42^,^43^. We hypothesize that under conditions where ATP levels are lowered due to reduction in ATP synthase activity, *M. tuberculosis* can utilize accumulated PolyP as phosphate donor for synthesis of ATP. This PolyP dependent ATP synthesis might be important for reactivation of *M. tuberculosis* from dormancy. The results presented in this and other studies demonstrate that PolyP metabolic enzymes are essential for *M. tuberculosis* growth in guinea pigs and are promising *in vivo* drug targets for identification of novel scaffolds^28^,^29^,^34^. Although numerous studies have validated these enzymes as drug-targets, to the best of our knowledge this is the first study where high throughput screening has been performed to identify novel PPK-2 inhibitors. Using 96-well luciferase based assay system we have identified two scaffolds NSC- 9037 and NSC-35676 as primary lead compounds that are non-cytotoxic and specific in their ability to inhibit PPK-2 enzyme *in vitro* in a dose dependent manner. These scaffolds might be also useful to combat infections caused by other bacterial pathogens such as *V. cholerae* and *P. aeruginosa* that encodes for PPK-2 protein with considerable homology to *M. tuberculosis* protein.

In summary, we report that PPK-2 is required for *M. tuberculosis* growth during acute and chronic stage of infection. The identified PPK-2 specific scaffolds can be explored further to design structural analogs that are non-cytotoxic and are more potent against *M. tuberculosis* PPK-2 enzyme *in vitro*. These results demonstrate the feasibility of 96-well luciferase based assay system to screen more libraries for identification of bacterial polyphosphate kinases inhibitors.

## Methods

### Bacterial strains and growth conditions

Bacterial strains and plasmids used in this study are listed in Table 1. *E. coli* strains were cultured in Luria-Bertani (LB) Miller medium. The mycobacterial strains were cultured in Middlebrook 7H9 broth (Difco, Becton, NJ, USA) supplemented with 10% albumin dextrose saline, 0.2% glycerol and 0.05% Tween-80 or in Middlebrook 7H11 agar plates at 37 °C as previously described^29^. The *in vitro* growth characteristics of various mycobacterial strains in the study was determined by measuring either absorbance at 600 nm or by plating 10-fold serial dilutions on MB 7H11 plates. The antibiotics were used at the following concentrations; ampicillin, 100 μg/ml; kanamycin (50 μg/ml for *E. coli* and 25 μg/ml for mycobacteria) and hygromycin B (150 μg/ml for *E. coli* and 50 μg/ml for mycobacteria). Standard protocols were followed for routine molecular biology experiments.

### Multiple sequence alignment studies

PPK-2 protein sequences from various microorganisms were retrieved from the National Centre of Biotechnology Institute (NCBI) protein database. Multiple sequence alignment was performed using Clustal omega software (version 1.2.0, http://www.ebi.ac.uk/Tools/msa/clustalo/) and edited using Genedoc software version 2.7.000^44^.

### Biochemical characterization of *M. tuberculosis* PPK-2 enzyme

For biochemical characterization, 888 bp region encoding for PPK-2 was PCR amplified using gene specific primers and cloned into a prokaryotic expression vector, pMAL-c2x (New England Biolabs, Ipswich, MA, USA). The primers used in this study are listed in Table S2. The recombinant plasmid pMAL-*ppk-2* was transformed into *E. coli* TB-1 cells and recombinant protein was induced by 1 mM IPTG addition and purified using amylose resin as per manufacturer’s recommendations. The purified protein fractions were pooled, concentrated and stored in storage buffer (20 mM Tris-Cl, pH-7.4, 200 mM NaCl, 1 mM EDTA and 10% glycerol) till further use. For biochemical characterization, glycine 72, lysine 75, phenylalanine 125 and tryptophan 129 amino acid residues were mutated to alanine residue by overlap extension PCR as per standard protocols and mutant genes were cloned into pMAL-c2x. PPK-2 activity assays were performed in buffer containing 50 mM Tris-Cl, pH-7.4, 10 mM (NH4)_2_SO_4_, 10 mM MgCl_2_ and 1 μM purified PPK-2 proteins. The amount of ATP generated in the enzyme reaction was quantified using Bactitre glo reagent as per manufacturer’s recommendations (Promega, Madison, WI, USA).

### Real time PCR studies

For qRT-PCR studies, *M. tuberculosis* H_37_Rv strains were cultured till early-log phase and subsequently exposed to various stress conditions and drugs as described previously^29^. mRNA was isolated by bead beating, purified using RNAeasy column and DNase I treated using Ambion DNA-free kit (Invitrogen Corporation, Carlsbad, CA, USA). 1 μg of Dnase I treated mRNA was used for cDNA synthesis using Superscript III reverse transcriptase. *In vitro* synthesized cDNA was used as template for quantitative PCR using gene specific primers. The primer sequences used for qRT-PCR studies have been listed in Table S2. The cycle threshold (*C*_*T*_) obtained for each gene of interest was normalized with that of *sigA*, housekeeping gene in order to obtain a normalized *C*_*T*_ value. The transcript levels of *ppk-2* neighboring genes (Rv3231c, Rv3233c and Rv3234c) and enzymes involved in PolyP metabolism (*ppk-1, ppk-2, ppx-1 and ppx-2*) were measured and compared in both wild type and *ppk-2* mutant strain at mid-log, late-log and stationary phase growth conditions.

### Construction of *ppk-2* mutant and complemented strains

Primer pairs *ppk-2* UpF/UpR and *ppk-2* DnF/DnR were designed to generate 800 bp upstream and downstream amplicons, respectively. These amplicons were gel eluted and cloned into cosmid vector pYUB854 flanking hygromycin resistance gene^33^. The recombinant cosmid pYUB854-Δ*ppk2* was packaged into phagemid phAE-159 using Gigapack III packaging extract (Agilent Technologies, Santa Clara, CA, USA). The *ppk-2* mutant strain of *M. tuberculosis* was constructed by homologous recombination using these temperature sensitive mycobacteriophages as per standard protocols^33^. The replacement of open reading frame for PPK-2 with hygromycin resistance gene in *ppk-2* mutant strain was confirmed by Southern blot and qRT-PCR analysis. For construction of *ppk-2* complemented strain, *ppk-2* DNA fragment was PCR amplified and cloned in mycobacterial shuttle integrative vector pJEB402^45^. The recombinant plasmid, pJEB-*ppk-2* was electroporated into *ppk-2* mutant strain and restoration of *ppk-2* expression in the mutant strain was confirmed by qRT-PCR. The primer sequences for *ppk-2* mutant and *ppk-2* complemented strain construction are listed in Table S2.

### Growth kinetics and *in vitro* stress experiments

*M. tuberculosis* wild type, *ppk-2* mutant and *ppk-2* complemented strains were cultured in MB 7H9 medium and their growth characteristics were monitored by measuring CFU/ml. For *in-vitro* stress experiments various strains were cultured, harvested at OD_600nm_ 0.3–0.4, washed with MB7H9 medium and exposed to either oxidative stress (5 mM H_2_O_2_) for 24 hours or nitrosative stress (5 mM NaNO_2_) for 72 hours or nutritional stress (1x tris buffered saline-tween-80) for 7 days as per standard protocols^29^. For *in vitro* drug tolerance, various strains were grown till mid-log stage (OD_600nm_ 1.5–2.0) and subsequently exposed to drugs with different mechanism of action such as Rif (transcriptional inhibitor), Levo (replication inhibitor), Inh (cell wall inhibitor) and Gm (translational inhibitor). After 14 days of drug-exposure, bacilli were harvested, washed twice with antibiotic free medium and resuspended in 1 ml of MB7H9 medium. The enumeration of viable bacteria was performed by plating 100 μl of 10-fold serial dilutions on MB7H11 plates in duplicates. For electron microscopy analysis, various strains were grown till late-log phase, fixed and stained with alcoholic uranyl acetate and alkaline lead citrate as per standard protocols^29^. The electron microscopy images were captured using Morgagni 268D transmission electron microscope (Fei company, Hillsbro, OR, USA). For MIC_99_ determination, various drugs were tested against wild type and *ppk-2* mutant strain from 50 μM to 0.04875 μM concentration in 96-well plates as per standard protocols. The growth inhibition in the presence of drugs was measured macroscopically using an inverted plate reader method at least two independent times.

### Infection of THP-1 macrophages with *M. tuberculosis*

THP-1 cells were maintained at 37 °C, 5% CO_2_ in RPMI medium supplemented with 10% fetal bovine serum (FBS, Invitrogen Corporation, Carlsbad, CA, USA). THP-1 monocytes were differentiated into macrophages by overnight addition of 15 nM phorbol-12-myristate-13-acetate (PMA). Macrophages were seeded at a cell density of 2 × 10^5^ in a 12 well plate and infected with various *M. tuberculosis* strains at an MOI of 1:1. After 4 hours post-infection, macrophages were washed and extracellular bacteria were removed by overlaying macrophages with RPMI medium containing 200 μg/ml amikacin for 2 hours. Subsequently, macrophages were washed twice with antibiotic free medium and overlayed with RPMI medium supplemented with 10% FBS. At days 0, 2, 4 and 6 post-infection, macrophages were lysed in 1× PBS containing 0.1% Triton X-100 and bacterial enumeration was done as described above.

### *In vivo* guinea pig experiments

Protocols for the animal experiments were reviewed and approved by the Institutional Animal Ethics Committee of International Centre for Genetic Engineering and Biotechnology (ICGEB/AH/2012/TACF/THSTI-2). Pathogen free out-bred female guinea pigs (Duncan Hartley strain, weight 300–350 g) were purchased from Lala Lajpat Rai University of Veterinary and Animal Sciences, Hisar, India. Guinea pigs were housed at Tuberculosis Aerosol challenge facility according to the procedures of the ICGEB animal care committee. For animal experiments, guinea pigs were infected via aerosol route using a Madison aerosol chamber with approximately 100 CFU of either *M. tuberculosis* H_37_Rv or *ppk-2* mutant or *ppk-2* complemented strain. Guinea pigs (n = 6/7) were sacrificed at 28 days and 56 days post-infection for determination of lung and splenic bacillary loads as described previously^29^. For histological studies, portion of lungs were fixed in 10% formalin, embedded in paraffin wax and stained with hematoxylin and eosin. The embedded tissues were coded, the extent of tissue damage and cellular infiltration was analysed by a pathologist as described previously^29^. The tissue samples were coded and evaluation of lung sections for granulomatous organization was performed by a pathologist having no prior knowledge of the experimental groups. The whole section of lung was examined and the number of granulomas were counted. Each granuloma was graded by the criteria as described previously^29^,^46^. Briefly, granulomas with necrosis were given a score of 5, granulomas with centrally placed epitheloid cells but no necrosis were given a score of 2.5 and granulomas with fibrous connective tissue surrounding lymphocytes and epitheloid cells were given a score of 1. Total granuloma score was obtained by multiplying number of granulomas of each type by the score and then adding them up to obtain a total granuloma score for each sample.

### High throughput screening to identify PPK-2 inhibitors

The luciferase based assay system was adapted to a 96-well format to identify novel PPK-2 small molecule inhibitors. For inhibition studies, an endpoint enzyme reaction was performed in assay conditions as described earlier in the absence and presence of inhibitor. The compounds screened belonged to small molecule library procured from National Cancer Institute–Developmental Therapeutic Program (NCI-DTP; http://dtp.nci.nih.gov/repositories.html). NCI-DTP library consists of 2300 compounds belonging to diversity set, mechanistic set or natural product set. Preliminary screening was performed at 100 μM concentration and all reaction plates included controls such as no buffer, no substrate, no enzyme and complete reaction control. For inhibition studies, PPK-2 enzyme was pre-incubated with small molecules for 10 minutes at 37 °C and the enzyme reaction was initiated by addition of 10 μM of PolyP_17_. The amount of ATP generated in enzymatic reactions was measured and percentage inhibition for each compound was calculated and plotted. Half-maximal inhibitory (IC_50_) concentration for identified primary hits (hits inhibiting PPK-2 activity by >50%) was determined by performing activity assays in the presence of increasing concentration of compounds in triplicates. The cellular cytotoxicity (TC_50_) for these primary hits against THP-1 macrophages was determined as described previously using Cell Proliferation WST-1 kit as per manufacturer’s recommendations^47^ (Sigma Aldrich, St. Louis, MI, USA).

### Statistical analysis

Graph Pad Prism 5 software (version 5.01, GraphPad Software Inc., CA) was used for the generation of graphs. Differences were considered statistically significant with P < 0.05.

### Ethics statement

This study was carried out in accordance with the guidelines provided by committee for the purpose of control and supervision on experiments on animals (CPCSEA, Govt. of India). The animal experiments were approved by the Animal ethics committee of the International Centre for genetic engineering and biotechnology, New Delhi, India (Approval No:- ICGEB/AH/2012/1/TACF-THSTI-2).

## Additional Information

**How to cite this article**: Singh, M. *et al* Establishing Virulence Associated Polyphosphate Kinase 2 as a drug target for *Mycobacterium tuberculosis*. *Sci. Rep.* **6**, 26900; doi: 10.1038/srep26900 (2016).

## Supplementary Material

Supplementary Information

## Figures and Tables

**Table 1:** List of Strains and Plasmids used in this study.

Strains	Description	Reference
XL-1 Blue	*recA1 endA1 gyrA96 thi-1 hsdR17 supE44 relA1 lac* [*F´ proAB lacIq Z*∆*M15 Tn10* (*Tetr*)]	Stratagene, USA
HB-101	*F-, thi-1, hsdS20* (*r*_*B*_ − *m*_*B*_)*, supE44, recA13, ara-14, leuB6, proA2, lacY1, galK2, rpsL20* (*strr*)*, xyl-5, mtl-1*	Promega, USA
BL-21(λDE3), plysS	*F-, ompT, hsdS*_*B*_ (*r*_*B*_ − *m*_*B*_)*, dcm, gal, λ*(*DE3*)*, plysS, cmr*	Promega, USA
TB-1	*F*^*−*^*ara*Δ(*lac-proAB*)[*ϕ80dlac*Δ (*lacZM15*)] *rpsL*(*Str*^*R*^)*thi hsdR*	New England Biolabs, USA
*mc*^*2*^*155*	*M. smegmatis* parental strain	A kind gift from Prof. Anil K Tyagi.
H_37_Rv	Laboratory Strain (ATCC 27294) of *Mtb*	ATCC
*ppk-2 mutant*	*ppk-2* mutant strain of *Mtb*	This study
*ppk-2 complemented*	*ppk-2::hyg* complemented with *ppk-2* at attB site	This study
Plasmids		
pYUB854	Cloning vector, hyg^R^	33
pYUB-Δ*ppk-2*	pYUB854 vector carrying upstream and downstream regions of *ppk-2*, hygromycin cassette	This study
phAE159	Phagemid DNA, Amp^R^	33
phAE159-Δ*ppk-2*	Phagemid DNA carrying upstream and downstream regions of *ppk-1*, hygromycin cassette	This study
pJEB-402	*E. coli* mycobacterium shuttle vector, Kan^R^	45
pJEB402-*ppk-2*	pJEB402 carrying *Rv3232c*	This study
pMAL-c2x	Prokaryotic Expression vector	New England Biolabs
pMAL-*ppk-2*	pMAL-c2x carrying PPK-2	This study
pMAL-G72A *ppk-2*	pMAL-c2x carrying PPK-2 with glycine 72 to alanine mutation	This study
pMAL-K75A *ppk-2*	pMAL-c2x carrying PPK-2 with lysine 75 to alanine mutation	This study
pMAL-F125A *ppk-2*	pMAL-c2x carrying PPK-2 with phenylalanine 125 to alanine mutation	This study
pMAL-W129A *ppk-2*	pMAL-c2x carrying PPK-2 with Tryptophan 129 to alanine mutation	This study
