# Author Correction: Establishing Virulence Associated Polyphosphate Kinase 2 as a drug target for *Mycobacterium tuberculosis*

**DOI:** 10.1038/s41598-020-72745-6

**Published:** 2020-09-21

**Authors:** Mamta Singh, Prabhakar Tiwari, Garima Arora, Sakshi Agarwal, Saqib Kidwai, Ramandeep Singh

**Affiliations:** Vaccine and Infectious Disease Research Centre, Translational Health Science and Technology Institute, Faridabad, Haryana India

Correction to: *Scientific Reports* 10.1038/srep26900, published online 9 June 2016

This Article contains an error in Figure 4a where the representative lung image for the wild type infected animals at 8 weeks is incorrect. The correct Figure 4a appears below as Figure 1.

**Figure 1:**
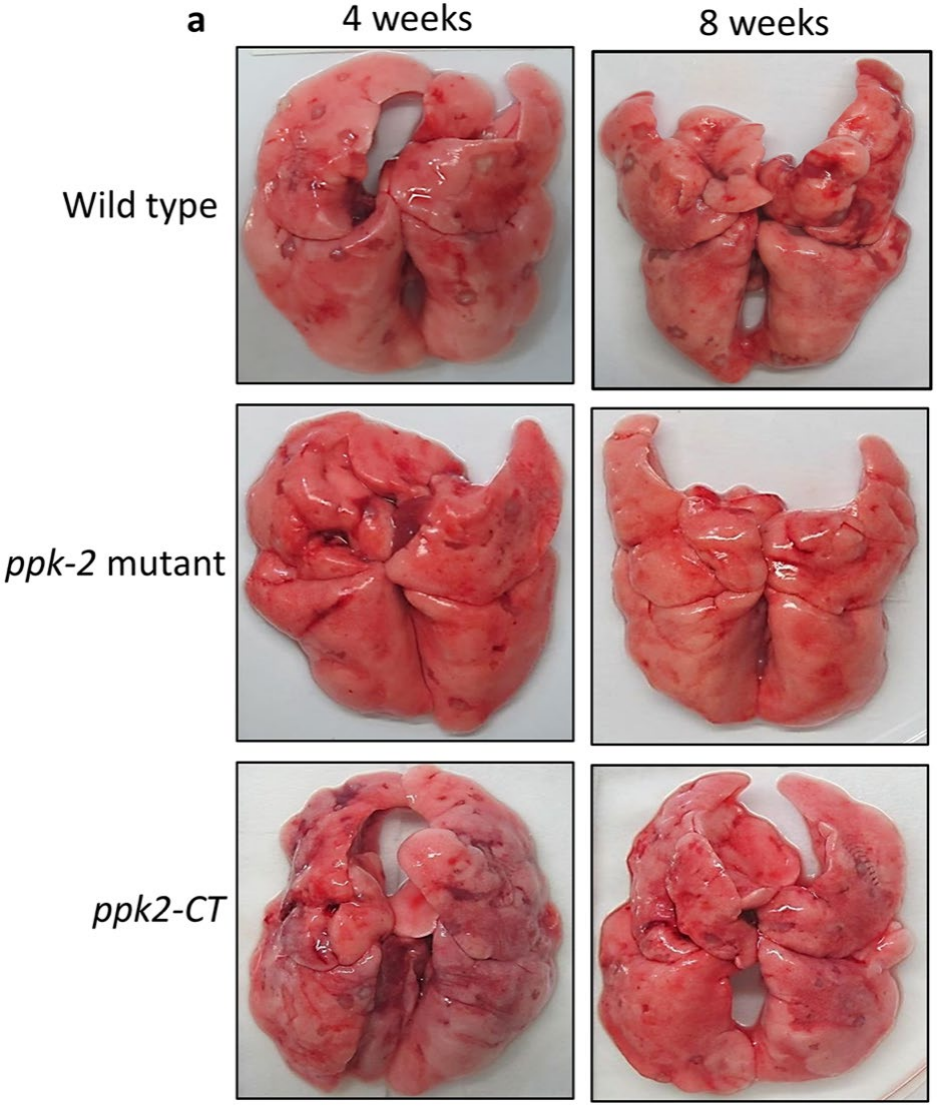
The panel depicts representative lung images from 4 weeks or 8 weeks infected guinea pigs.

