# Author Correction: Establishing Virulence Associated Polyphosphate Kinase 2 as a drug target for *Mycobacterium tuberculosis*

**DOI:** 10.1038/s41598-021-00316-4

**Published:** 2021-11-02

**Authors:** Mamta Singh, Prabhakar Tiwari, Garima Arora, Sakshi Agarwal, Saqib Kidwai, Ramandeep Singh

**Affiliations:** Vaccine and Infectious Disease Research Centre, Translational Health Science and Technology Institute, Faridabad, Haryana India

Correction to: *Scientific Reports* https://doi.org/10.1038/srep26900, published online 09 June 2016

This Article contains errors.

The data used for Supplementary Fig. 2B is incorrect. We have repeated this experiment, and the new data are presented below.

**Figure:**
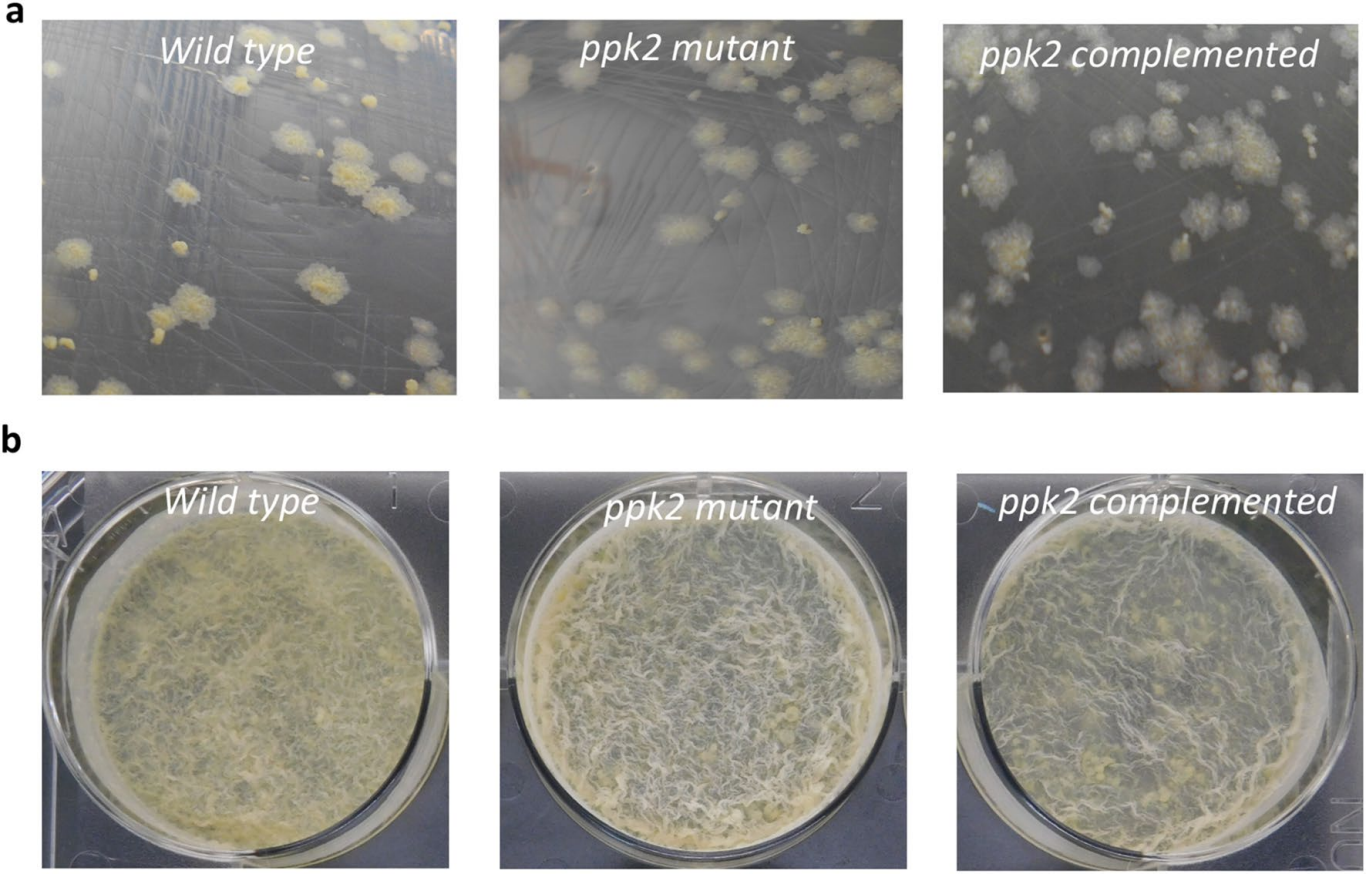

**Supplementary Fig. 2.** **Effect of PPK-2 deletion on colony morphology (A) and biofilm formation (B) of** ***M. tuberculosis***. (A) Colony morphology of various *M. tuberculosis* strains was determined by plating 10-fold serial dilutions on MB-7H11 plates. (B) Biofilm images of the standing cultures of wild type, *ppk-2* mutant and *ppk-2* complemented strains in polystyrene coated 6-well plates in Sauton’s medium. The images depicted are representative of two independent experiments.

