# Supplementary material for: Establishing Virulence Associated Polyphosphate Kinase 2 as a drug target for Mycobacterium tuberculosis
Source: Sci Rep. 2016 Jun 9;6:26900. doi: 10.1038/srep26900 (PMC4899718; doi:10.1038/srep26900)
Supplement: Supplementary Information [file srep26900-s1.pdf]

Establishing Virulence Associated Polyphosphate Kinase 2 as a drug target for *Mycobacterium tuberculosis*

Mamta Singh, Prabhakar Tiwari, Garima Arora, Sakshi Agarwal, Saqib Kidwai and Ramandeep Singh*

Vaccine and Infectious Disease Research Centre, Translational Health Science and Technology Institute, Haryana, India.

Running title: *Targeting polyphosphate kinase-2 of M. tuberculosis*

*Corresponding author. Mailing address: NCR Biotech Science Cluster, 3rd Milestone, Faridabad-Gurgaon Expressway. PO Box # 4. Faridabad – 121001.

Keywords: *Mycobacterium tuberculosis*, inorganic polyphosphate, virulence and high through put screening

Supplementary Fig. 1: Multiple Sequence Alignment studies. The multiple sequence alignment among PPK-2 protein sequences from various microorganisms was performed using Clustal Omega (version 1.2.0) and using Gene doc. Black, dark grey and clear gray shaded areas in the alignment indicate 100%, 80% and 60% conservation, among these proteins respectively. The protein sequences from following microorganisms were selected for alignment studies; MS represents PPK-2 from *Mycobacterium smegmatis* mc^2 155; PA1, PA2 represents PA0141, PA2428, of *P. aeruginosa* PAO1 respectively; SM1, SM2 and SM3 represents SMa0172, SMa0670, and SMc02148 of *Sinorhizobium meliloti* 1021 respectively; MT represents Rv3232c of *Mycobacterium tuberculosis* $H_{37}Rv$; RP1, RP2, RP3 indicates SPO1727, SPO1256 and SPO0224 *Ruegeria pomeroyi* DSS-3, respectively.

Supplementary Fig. 2: Effect of PPK-2 deletion on colony morphology (A) and biofilm formation (B) of *M. tuberculosis*. (A) Colony morphology of various *M. tuberculosis* strains was determined by plating 10-fold serial dilutions on MB-7H11 plates.

(B) Biofilm images of the standing cultures of wild type, *ppk-2* mutant and *ppk-2* complemented strains in polystyrene coated 6-well plates in Sauton's medium. The images depicted are representative of two independent experiments.

Table S1: MIC values of various drugs against *M. tuberculosis* wild type and *ppk-2* mutant strains.

Drugs	*M. tuberculosis* $H_{37}Rv$	*ppk-2 mutant*	*ppk-2 complemented*
Rifampicin	0.39 nM	0.39 nM	0.39 nM
Isoniazid	0.78 μM	0.78 μM	0.78 μM
Ethambutol	2.5 μM	2.5 μM	2.5 μM
Levofloxacin	0.78 μM	0.78 μM	0.78 μM
Chloramphenicol	1.95 μg/ml	1.95 μg/ml	1.95 μg/ml
Vancomycin	1.25 μg/ml	1.25 μg/ml	1.25 μg/ml
Gentamycin	1 μg/ml	1 μg/ml	1 μg/ml

Table S2: Sequence of primers used in the present study.

**Primers used for construction of various mutant and overexpression strains**			
	Forward (5’ ------ 3’)	Reverse (5’ ------- 3’)	
ppk-2 upstream	gggaggcctactcgtcgagacgggccgcaagg	gggtctagaggtgctcacatcaacggatggtatatcc	
ppk-2 downstream	ggaagcttgactatgtggccacgttgatcgcc	gggactagtccgcgcggaccacagcggatttgc	
ppk-2 complemented	gggaggcctcagtggatataccatccgttgatg	gggaagctttcaccgggcgatcaacgtggcc	
ppk-2 ORF	ggcatatggtggatataccatccgttgatgtgagc	gaagctttcaccgggcgatcaacgtggccac	
ppk-2 G72A	cgaaggccgtgacgcagctggtaagg	ccttaccagctgcgtcacggccttcg	
ppk-2 K75A	cggagctggtgcgggtggcgccatcaa	ttgatggcgccacccgcaccagctccg	
ppk-2 F125A	gagatagtgctcgccgatcggtcc	ggaccgatcggcgagcactatctc	
ppk-2 W129A	atcggtccgcgtacaaccgcgccg	cggcgcggttgtacgcggaccgat	
**Primers used for RT-PCR using SYBR green mix**			
	Forward primer (5’ ------ 3’)	Reverse primer (5’ ------ 3’)	
sigA	acgaagaccacgaagacctcgaa	gtaggcgcgaaccgagtcggcgg	
Rv3231c	ccgcagacgccctgccgcccagacg	ccgcaatcgccgccatcacggcc	
Rv3233c	aatccaacccgaccgccgccagc	tgcagtagcggcggcacggagtagg	
Rv3234c	cgacatcacctaccacgtccggcgg	ccccgttgatcagggcttggtgcga	
**Primers used for RT-PCR using Taqman probes**			
	Forward primer (5’----3’)	Reverse primer (5’---- 3’)	Taqman Probes (5’---- 3’)
sigA	gacgaggagatcgctgaacc	tcgtcttcatcccagacgaaa	ccgaaaaggacaaggcctccgg
ppk-1	cggcggtactgccatgtc	cagtccgacgtcctcgtagag	caccggcaattacaacagcaagacagc
ppk-2	acgcgcggctgaacat	ggcttttccacatctgcgtagt	atggcccacttgctgtccacgatt
ppx-1 (Rv0496)	ggaccctaacggcaaatggt	tccagttctgcacggtcaa	tgcggcaactcatcgcgtttatctcatctc
ppx-2 (Rv1026)	cagtccatgacggcgtatg	ggctaaacctcgagcagat	ctgcggccattcatctttcgcg

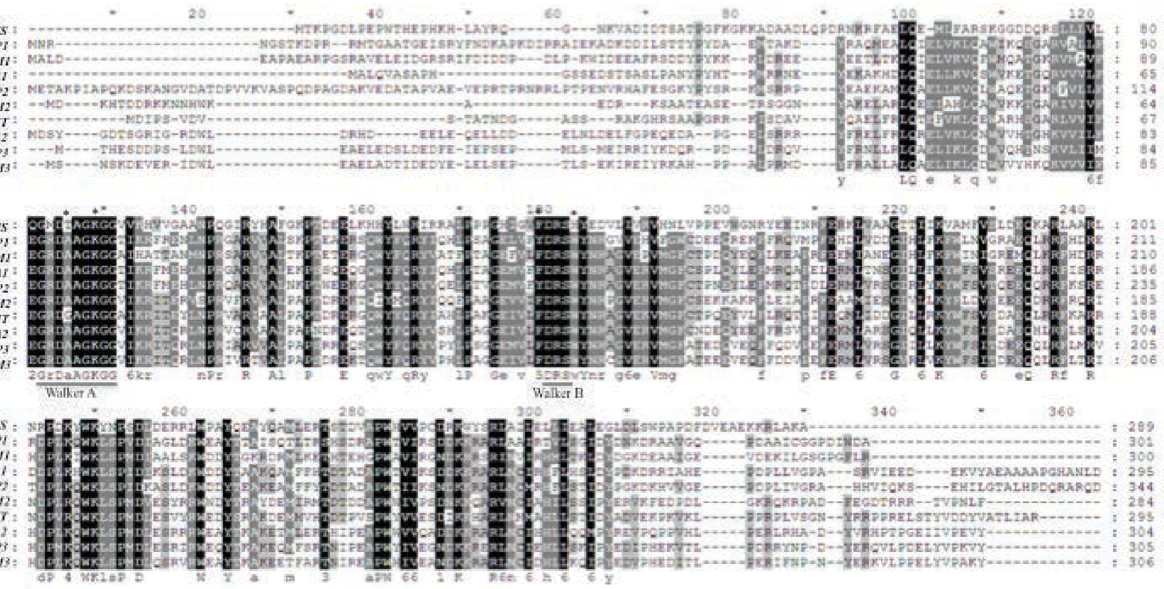
MS
RP1
SM1
PA1
RP2
SM2
MT
PA2
RP3
SM3
Walker A
Walker B

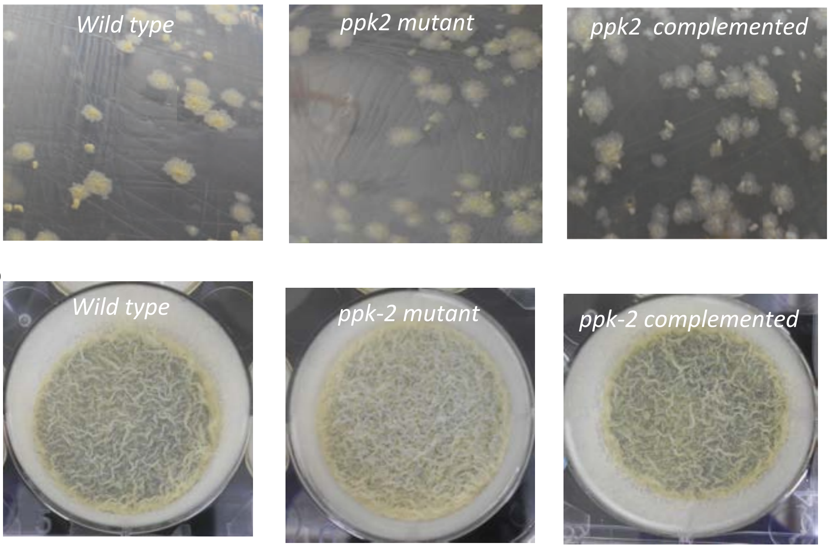
a
Wild type
ppk2 mutant
ppk2 complemented
b
Wild type
ppk-2 mutant
ppk-2 complemented
